# Biodiesel production from waste cooking oil using heterogeneous catalyst: Biodiesel product data and its characterization

**DOI:** 10.1016/j.dib.2019.104879

**Published:** 2019-11-23

**Authors:** Meilana Dharma Putra, Iryanti Fatyasari Nata, Chairul Irawan

**Affiliations:** Chemical Engineering Department, Faculty of Engineering, Lambung Mangkurat University, Indonesia

**Keywords:** Biodiesel, CaO, Silica, Peat clay, Waste cooking oil

## Abstract

The biodiesel production from waste cooking oil in this data collection process was focused on the utilization of the heterogeneous catalyst of CaO/silica. The CaO was obtained from eggshell after preparation process and the silica was successfully extracted from peat clay using sodium hydroxide with various molarities. The CaO/silica catalyst was formed by the impregnation of the CaO catalyst on the support of silica. The FTIR, SEM and XRD characterization for the various formed catalysts were presented. The generated catalysts were further used for the production of biodiesel. The GCMS chromatogram with the type of methyl esters for each data was presented. The data presented here are related to the previous research article [1].

**Table:** Specifications Table

Subject	Chemical Engineering
Specific subject area	Catalysis in Energy Process
Type of data	Tables and Figure
How data were acquired	SEM, FTIR, XRD and GCMS
Data format	Raw and analyzed
Experimental factors	The catalyst was characterized after activation (calcination) at 900 °C for 2 h. The transesterification reaction was conducted at the time range of 30–120 min and the temperature range of 30–60 °C.
Experimental features	The heterogeneous catalyst was collected from impregnation of CaO on silica support. The silica was obtained from extraction of peat clay.
Data source location	Lambung Mangkurat University, Banjarbaru, Indonesia
Data accessibility	Data were available within the article
Related research article	Meilana Dharma Putra, Chairul Irawan, Udiantoro, Yuli Ristianingsih and Iryanti Fatyasari Nata A cleaner process for biodiesel production from waste cooking oil using waste materials as a heterogeneous catalyst and its kinetic study Journal of Cleaner Production https://doi.org/10.1016/j.jclepro.2018.06.010

**Table:** 

**Value of the Data** • The similar characteristic observations for all developed catalysts are important to support the previous work for potential biodiesel production. • The researchers can explore other waste materials for biodiesel production development based on this finding. • The promising catalyst and the oil source from waste lead to the potential development for industrial application. • The type of methyl ester obtained was useful for characteristic and specific fuel desired.

## 1 Data

**Fig. 1:**
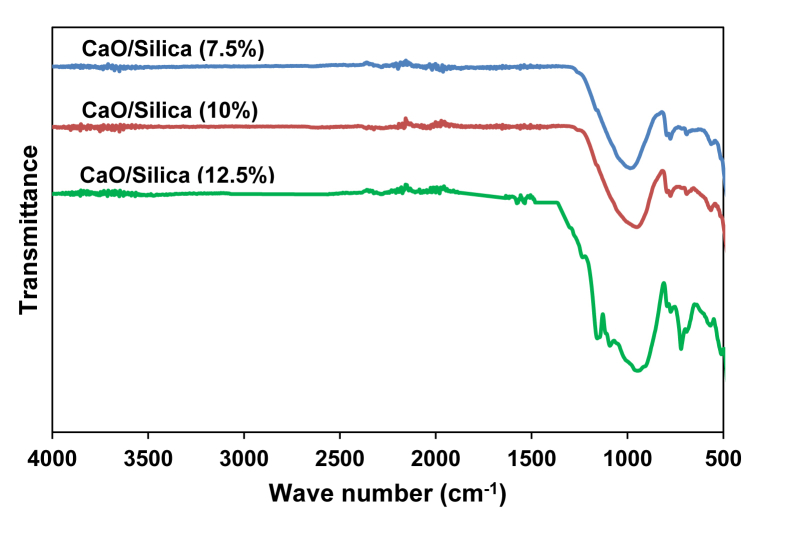
FTIR characterization for (a). CaO/silica (7.5%); (b). CaO/silica (10%) and (c). CaO/silica (12.5%).

**Fig. 2:**
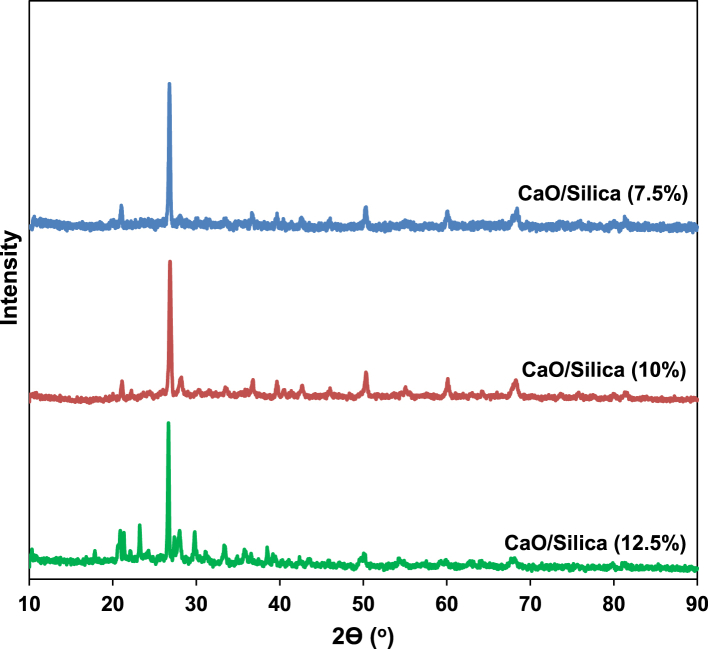
XRD characterization for (a). CaO/silica (7.5%); (b). CaO/silica (10%) and (c). CaO/silica (12.5%).

**Fig. 3:**
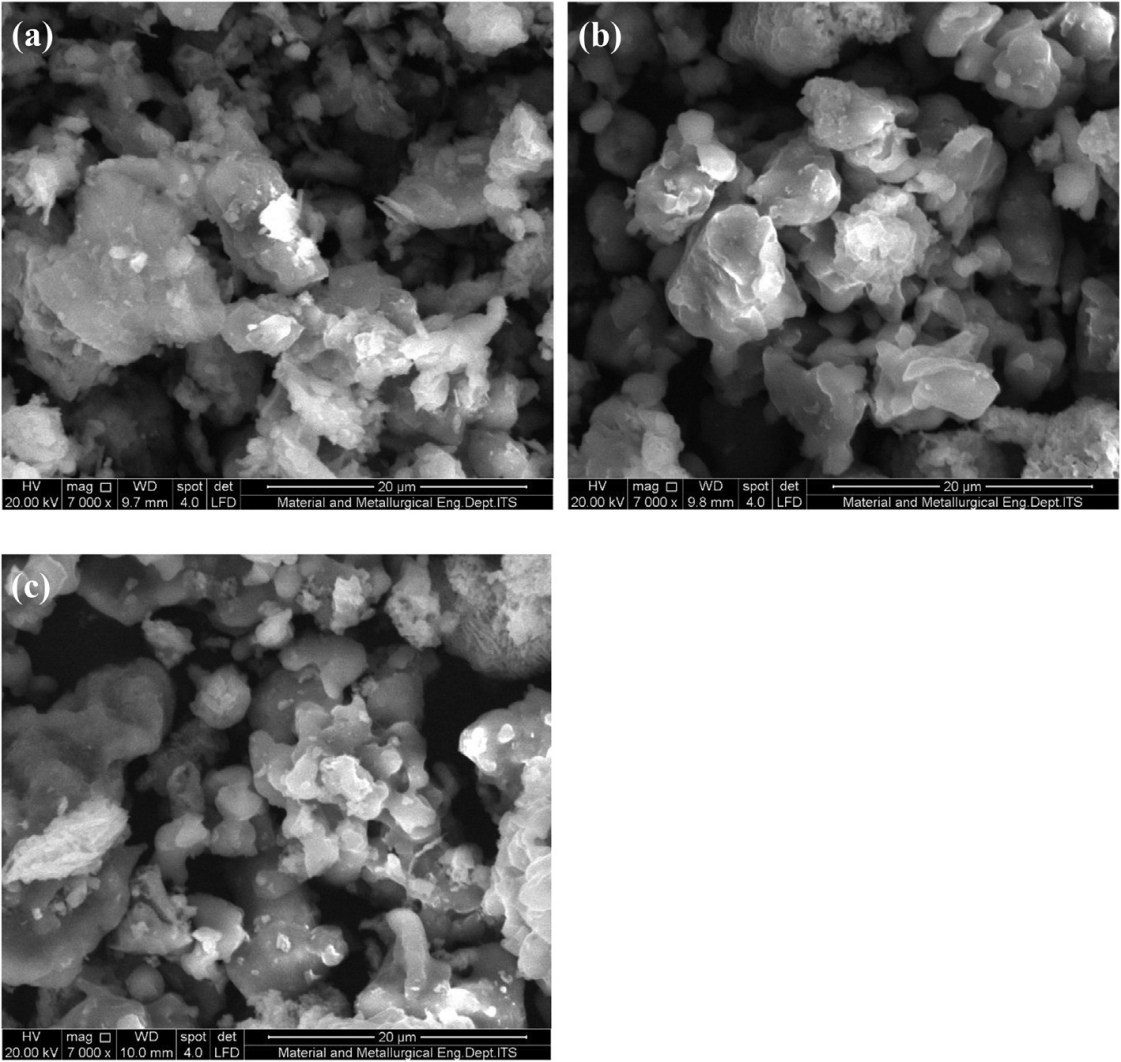
SEM characterization for (a). CaO/silica (7.5%); (b). CaO/silica (10%) and (c). CaO/silica (12.5%).

**Fig. 4:**
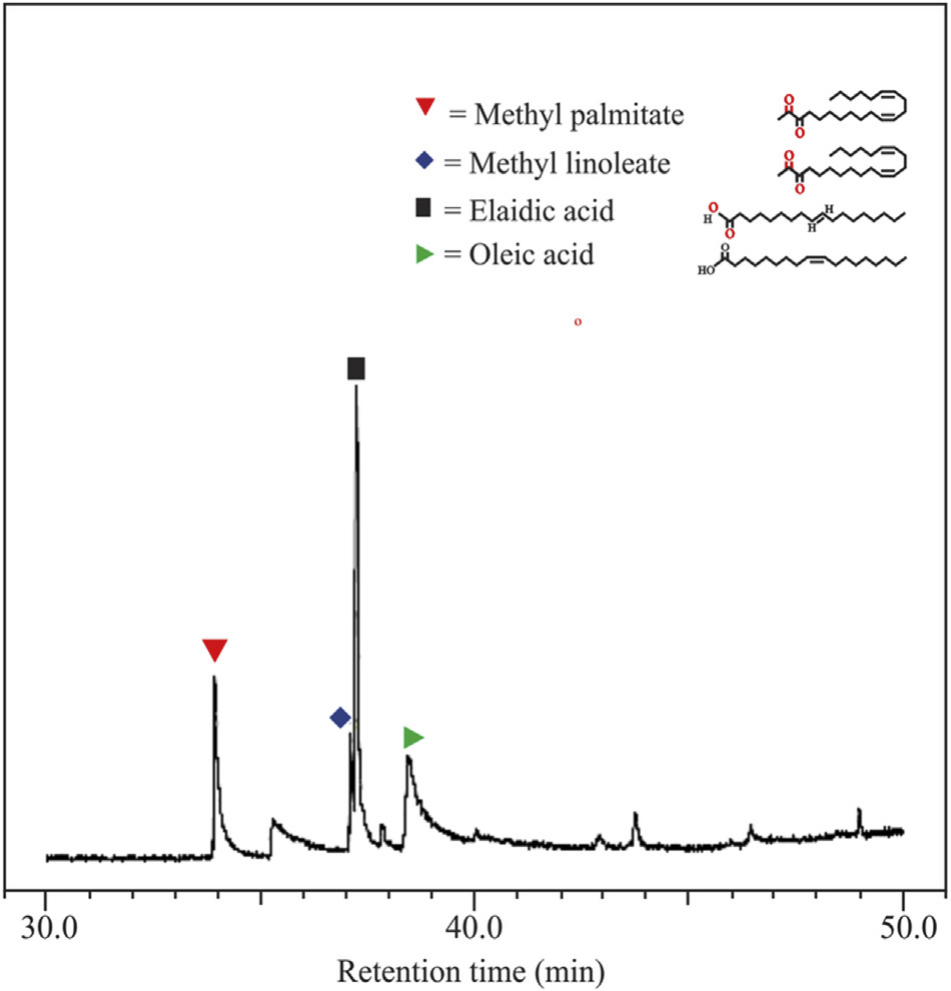
Chromatogram result of sample for 15 min at 60 °C with 14 M ratio of methanol to biodiesel.

**Fig. 5:**
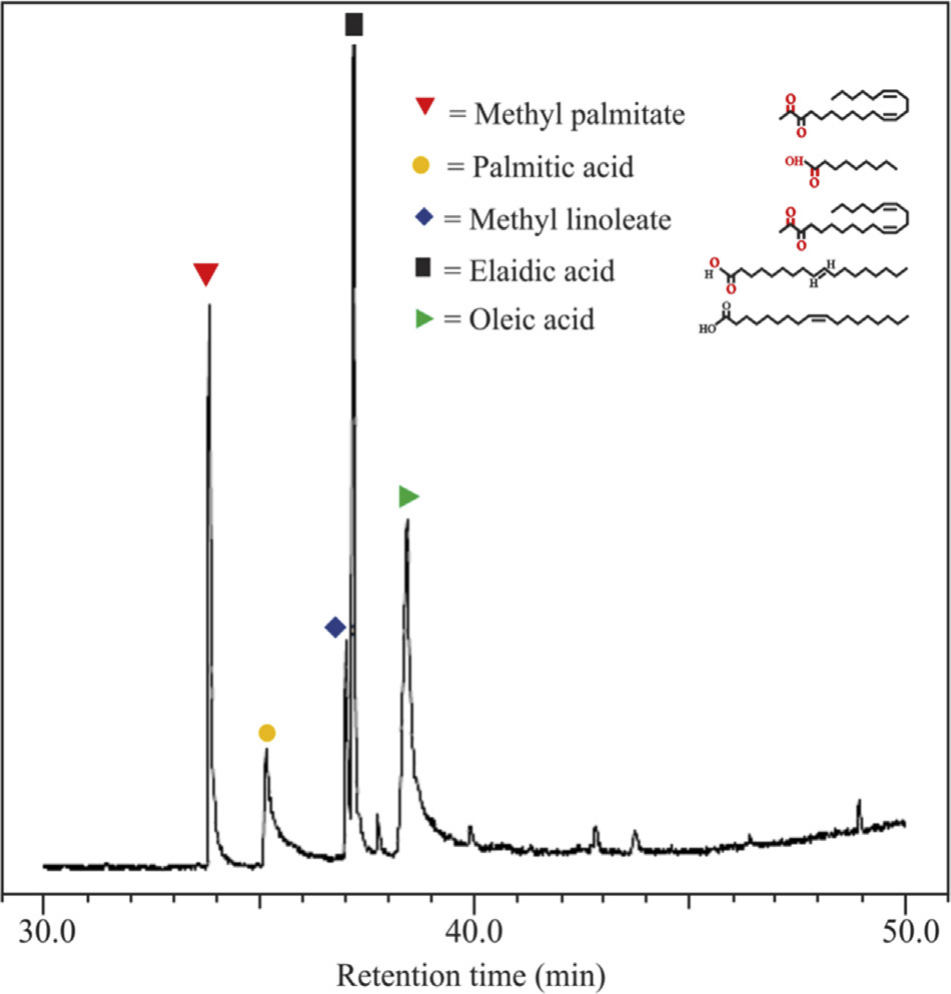
Chromatogram result of sample for 45 min at 60 °C with 14 M ratio of methanol to biodiesel.

**Fig. 6:**
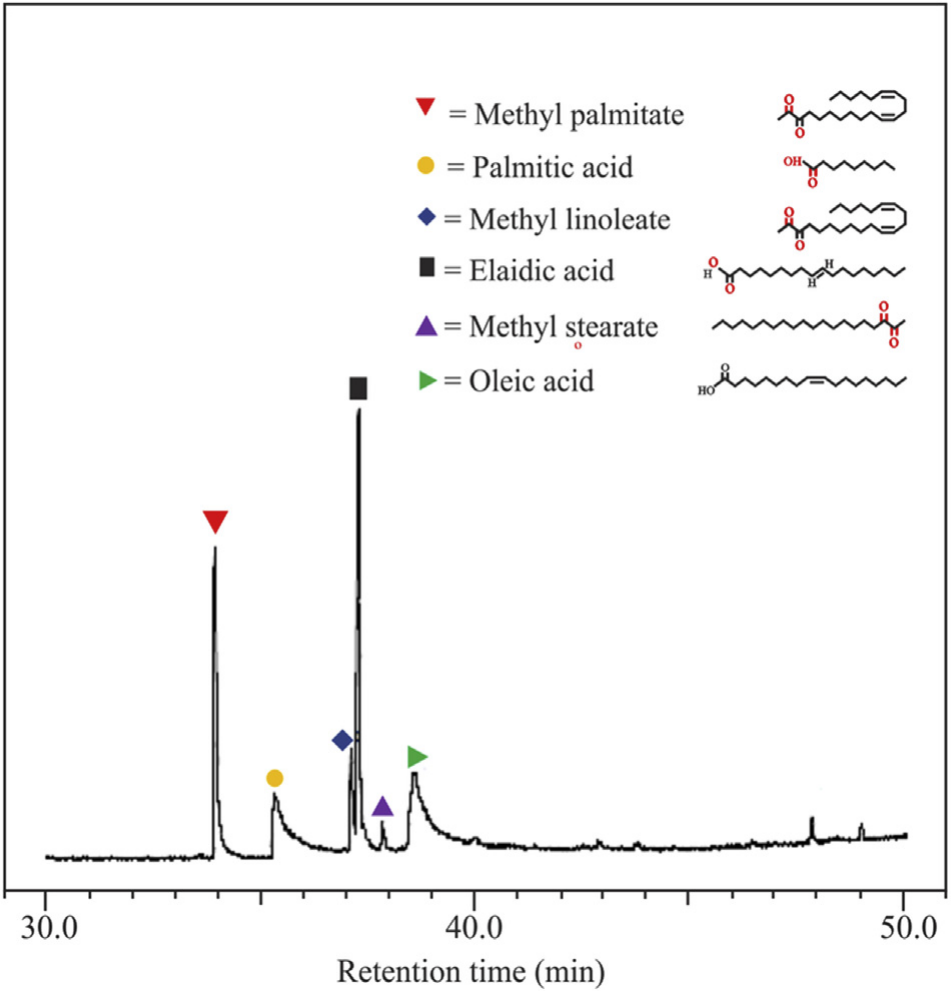
Chromatogram result of sample for 60 min at 60 °C with 14 M ratio of methanol to biodiesel.

**Fig. 7:**
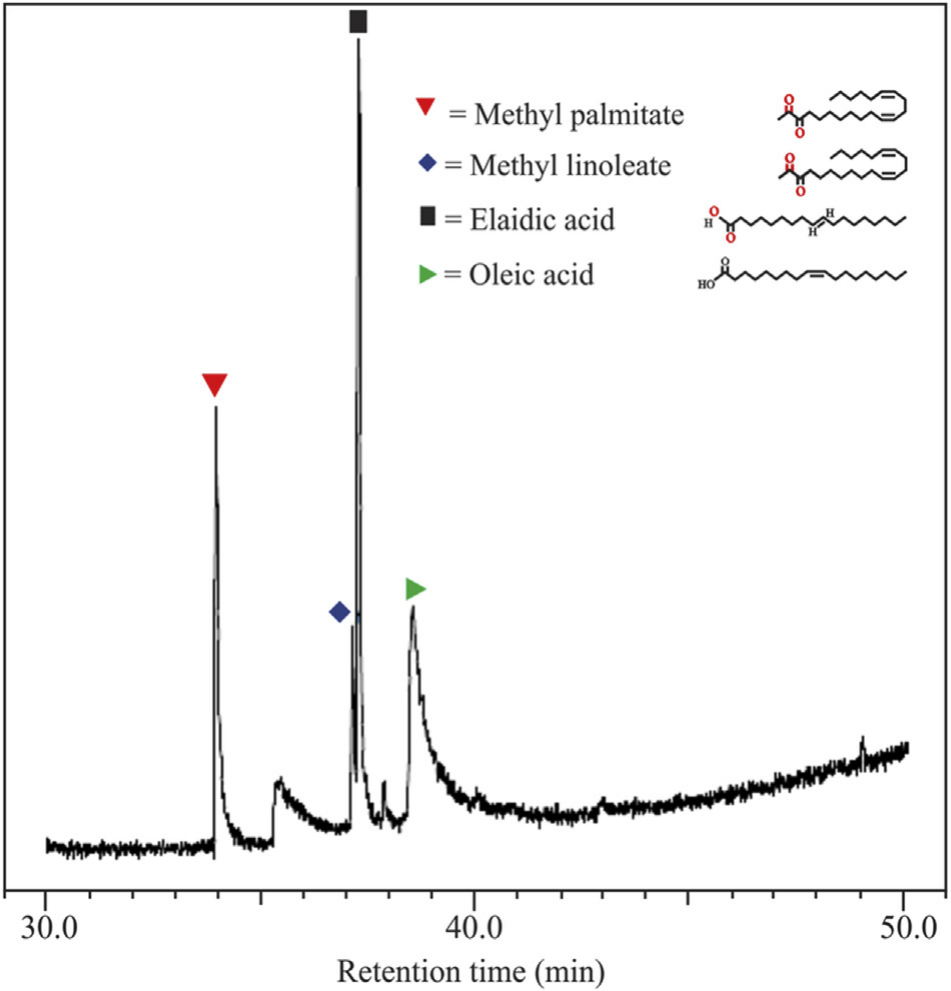
Chromatogram result of sample for 75 min at 60 °C with 14 M ratio of methanol to biodiesel.

**Fig. 8:**
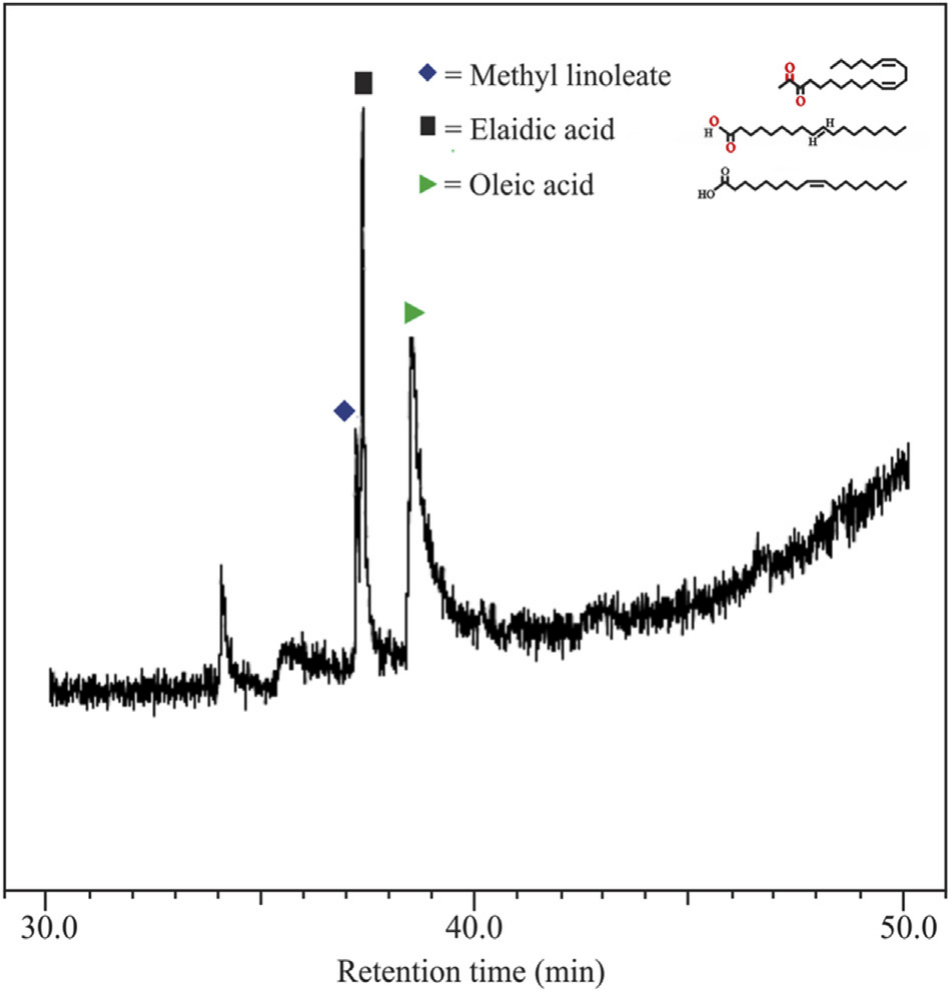
Chromatogram result of sample for 90 min at 60 °C with 21 M ratio of methanol to biodiesel.

**Fig. 9:**
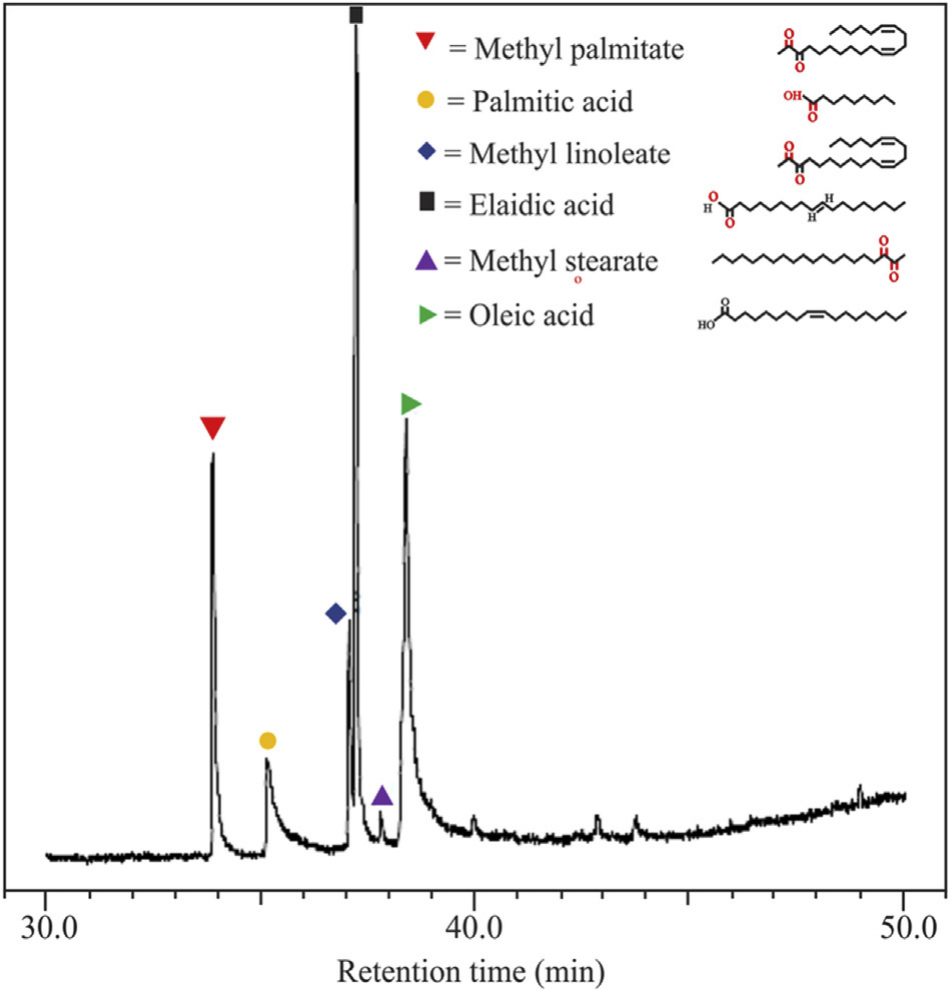
Chromatogram result of sample for 90 min at 60 °C with 17 M ratio of methanol to biodiesel.

**Fig. 10:**
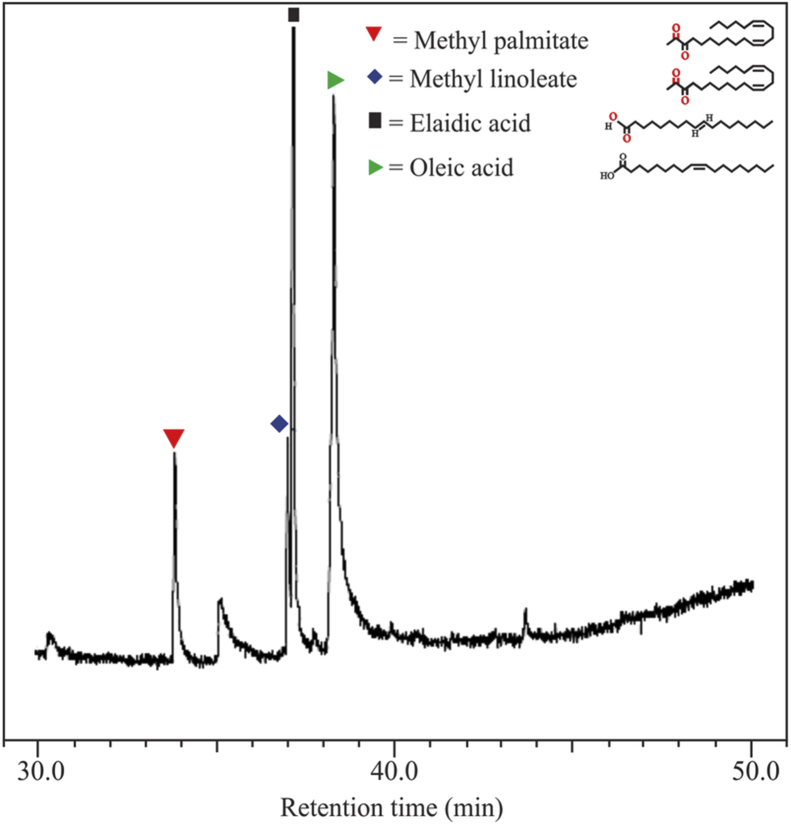
Chromatogram result of sample for 90 min at 60 °C with 12 M ratio of methanol to biodiesel.

Fig. 1 shows the FTIR characterization for three catalysts of CaO/silica. The similar observations were obtained for those catalysts; however, the strong peak was observed at 850 cm^−1^ and around 720 cm^−1^ for the type catalyst of CaO/silica (12.5%) due to Si–O interaction and CaO, respectively. Fig. 2 presents the XRD characterization for three catalysts of CaO/silica with the identical trends. The presence of CaCO3, CaO and silica was observed in the peak of around 30° [2], in the range of 34–44° [3] and about 27° [1], respectively. The similar observations were also shown for SEM characterization (with enlargement of 7000X) for the three types of CaO/silica catalysts as shown in Fig. 3. The important point of these finding is that the different concentrations of solvent for silica extraction lead to similar characteristic observations of the catalysts. Hence, the developed CaO/silica catalyst stands as a promising catalyst for biodiesel production. Fig. 4, Fig. 5, Fig. 6, Fig. 7, Fig. 8, Fig. 9, Fig. 10 show the chromatogram marks for various reaction condition. The components of methyl palmitate, methyl linoleate, elaidic acid and oleic acid were majorly observed as shown in Table 1. The composition of fatty acid was dominantly composed of the carbon chain of C16–C20 [4,5]. The different compositions of fatty acid resulted for various conditions are important for further development of this prospective work.

**Table 1:** Type of fatty acid component in biodiesel products.

Condition	Component					
	Methyl palmitate	Palmitic acid	Methyl linoleate	Elaidic acid	Methyl stearate	Oleic acid
T = 60 °C; t = 15 min; ratio = 14	25.24		10.77	49.37		13.93
T = 60 °C; t = 45 min; ratio = 14	24.87	4.99	8.39	8.39		29.18
T = 60 °C; t = 60 min; ratio = 14	28.04	7.88	8.22	35.90	2.58	16.98
T = 30 °C; t = 75 min; ratio = 14	29.54	0.43	8.71	38.79		22.23
T = 60 °C; t = 90 min; ratio = 21	0.54		22.49	64.24		12.03
T = 60 °C; t = 90 min; ratio = 18	17.18	6.21	8.54	32.39	0.90	34.68
T = 60 °C; t = 90 min; ratio = 12	11.91	0.35	9.20	28.36		49.86

## 2 Experimental design, materials, and methods

The catalyst of CaO was obtained from eggshell after process of cleaning, sieving and calcination was conducted. Silica as support of catalyst was extracted from peat clay upon the preparation process of cleaning, crushing, sieving and calcination were conducted. The extraction process was carried out at 80 °C for 1 h using 60 mL NaOH with the variation of molarities of 1.86 N, 2.5 N and 3.12 N. These molarities corresponded to the sodium hydroxide concentration of 7.5%, 10% and 12.5%. The CaO catalyst was finally combined with the obtained silica resulted from the extraction as support using impregnation method [6]. The three types of catalyst were then namely CaO/silica (7.5%), CaO/silica (10%) and CaO/silica (12.5%). The catalyst was utilized in the transesterification process at various temperature and time to produce biodiesel. The waste cooking oil and methanol were used as raw material. The biodiesel component was analyzed using GCMS (2010S Shimadzu, Tokyo, Japan). The catalyst was characterized using FTIR, XRD and SEM with the detailed equipment described there [1].
